# P-577. Improved Testing for Chronic Disease Among Long-Acting Injectable Versus Daily Oral Antiretroviral Therapy Users in the OPERA Cohort

**DOI:** 10.1093/ofid/ofae631.775

**Published:** 2025-01-29

**Authors:** Philip C Lackey, Rachel P Weber, Gerald Pierone, Michael G Sension, Anthony M Mills, Michael B Wohlfeiler, Jennifer S Fusco, Brooke Levis, Gayathri Sridhar, Vani Vannappagari, Jean A van Wyk, Gregory P Fusco

**Affiliations:** Wake Forest University School of Medicine, Winston Salem, North Carolina; Epividian, Inc., Raleigh, North Carolina; Whole Family Health Center, Vero Beach, FL; can community health, Miami Beach, FL; Men's Health Foundation, Los Angeles, CA, United States(LAX-Los Angeles International Airport), California; AIDS Healthcare Foundation, Miami Beach, Florida; Epividian, Inc., Raleigh, North Carolina; Epividian, Inc, Montreal, Quebec, Canada; ViiV Healthcare, Fairfax, Virginia; ViiV Healthcare, Fairfax, Virginia; ViiV Healthcare, Brentford, UK, Brentford, England, United Kingdom; Epividian, Inc., Raleigh, North Carolina

## Abstract

**Background:**

People with HIV (PWH) develop more chronic illnesses at younger ages. Frequent healthcare interactions can help with early detection and management of chronic disease. We aimed to describe the frequency and timing of testing related to chronic disease among PWH receiving long-acting (LA) antiretroviral therapy (ART) versus oral ART in the OPERA^®^ cohort.

**Figure:**
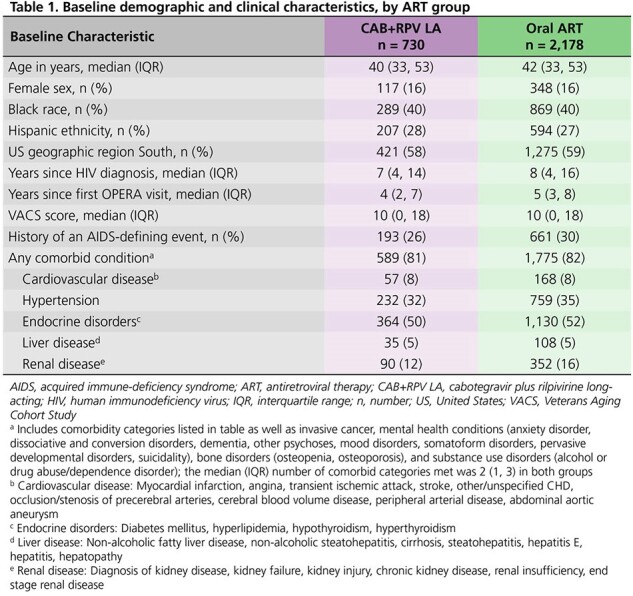

**Methods:**

We identified treatment-experienced, suppressed (viral load < 50 copies/mL) adults initiating cabotegravir plus rilpivirine (CAB+RPV) LA injections or a new oral ART regimen between 21JAN2021 and 30JUN2022. Each PWH initiating CAB+RPV LA was matched to 1-3 PWH initiating oral ART based on age, sex, and location. We followed matched groups until regimen discontinuation, death, loss to follow-up, or 30JUN2023 and calculated the proportions of PWH receiving metabolic testing and risk score calculations (ASCVD, FIB-4, eGFR, and VACS Mortality Index) over follow-up.

**Figure:**
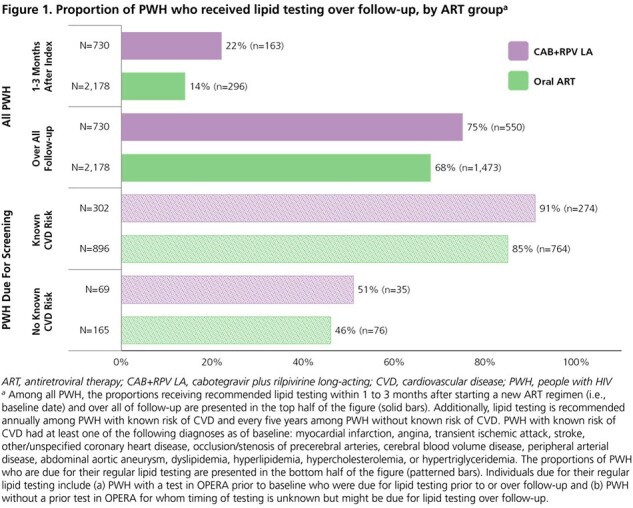

**Results:**

Baseline characteristics between 730 CAB+RPV LA and 2,178 oral ART users were similar (**Table 1**). Lipid (**Figure 1**) and serum glucose (**Figure 2**) testing occurred among a greater proportion of CAB+RPV LA than oral ART users over follow-up (lipids: 75% vs. 68%; glucose: 97% vs. 86%). Similarly, there were greater proportions of CAB+RPV LA PWH receiving those tests than oral ART PWH, among the subset of PWH who were due for those screenings. A smaller proportion of oral ART users received recommended lipid (14%) and glucose (34%) testing within 1-3 months after baseline compared to 22% and 52%, respectively, of CAB+RPV LA users. Risk scores were calculated among a greater proportion of CAB+RPV LA users (68-96%) than oral ART users (66-86%). Risk score components (usually lab values) were also updated sooner after baseline among CAB+RPV LA users, providing earlier opportunity for intervention as appropriate (**Table 2**).

**Figure:**
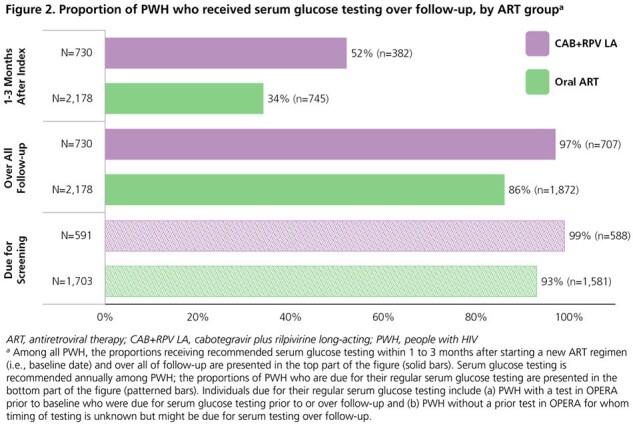

**Conclusion:**

Compared to oral ART users, a larger proportion of CAB+RPV LA users received recommended lipid and serum glucose testing; the same held true for risk score calculations related to mortality and cardiovascular, liver, and renal health, which also occurred sooner after baseline among CAB+RPV LA users. Regular clinic visits for CAB+RPV LA injections may provide an opportunity for earlier detection and management of chronic disease conditions.

**Figure:**
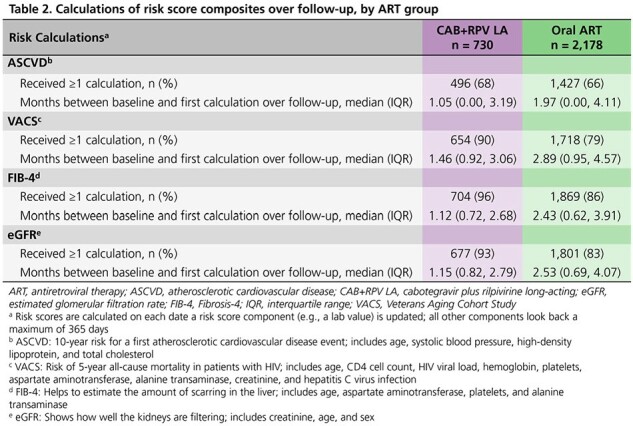

**Disclosures:**

**Rachel P. Weber, PhD**, EMD Serono: Research support to my employer|Gilead Sciences: Research support to my employer|Merck & Co.: Research support to my employer|TheraTechnologies: Research support to my employer|ViiV Healthcare: Research support to my employer **Gerald Pierone, Jr., MD**, GSK: Grant/Research Support|VIIV: Grant/Research Support **Michael G. Sension, MD**, Gilead: Grant/Research Support|Gilead: Honoraria|Viiv: Honoraria **Anthony M. Mills, MD**, Abbott: Grant/Research Support|Emit Bio: Grant/Research Support|Gilead: Advisor/Consultant|Gilead: Grant/Research Support|Merck: Advisor/Consultant|Merck: Grant/Research Support|ViiV Healthcare: Advisor/Consultant|ViiV Healthcare: Grant/Research Support **Jennifer S. Fusco, BS**, EMD Serono: Research support to my employer|Gilead Sciences: Research support to my employer|Merck & Co.: Research support to my employer|TheraTechnologies: Research support to my employer|ViiV Healthcare: Research support to my employer **Brooke Levis, PhD**, EMD Serono: Research support to my employer|Gilead Sciences: Research support to my employer|Merck & Co.: Research support to my employer|TheraTechnologies: Research support to my employer|ViiV Healthcare: Research support to my employer **Gayathri Sridhar, MBBS, MPH, PhD**, GlaxoSmithKline: Stocks/Bonds (Public Company)|ViiV Healthcare: Full Time Employee **Vani Vannappagari, MBBS, MPH, PhD**, GSK: Stocks/Bonds (Public Company)|ViiV Healthcare: Full time Employee|ViiV Healthcare: Stocks/Bonds (Public Company) **Jean A. van Wyk, MBChB, MFPM**, ViiV Healthcare: Employee|ViiV Healthcare: Stocks/Bonds (Public Company) **Gregory P. Fusco, MD, MPH**, EMD Serono: Research support to my employer|Gilead Sciences: Research support to my employer|Merck & Co.: Research support to my employer|TheraTechnologies: Research support to my employer|ViiV Healthcare: Research support to my employer

